# A comparison of robot-assisted thoracic surgery and median sternotomy for the treatment of early-stage thymoma: a propensity score matching study

**DOI:** 10.1007/s11748-026-02294-8

**Published:** 2026-04-18

**Authors:** Hirofumi Takenaka, Tetsuya Mizuno, Keita Nakanishi, Yuka Kadomatsu, Harushi Ueno, Taketo Kato, Shota Nakamura, Toyofumi Fengshi Chen-Yoshikawa

**Affiliations:** Department of Thoracic Surgery, Nagoya University Graduate School of Medicine, 65 Tsurumai-cho, Showa-ku, Nagoya, 466-8550 Japan

**Keywords:** Thymoma, Median sternotomy, Robot-assisted thoracic surgery, Thymectomy, Early-stage

## Abstract

**Objective:**

For early-stage thymoma, Robot-assisted approaches for early-stage thymomas have become widespread. This study aimed to compare the surgical and survival outcomes of median sternotomy and robot-assisted thoracic surgery in patients with early-stage thymoma.

**Methods:**

Between 2010 and 2024, 203 patients with Masaoka stage I or II thymoma underwent either robot-assisted thoracic surgery (n = 68) or median sternotomy (n = 135). The clinical characteristics, surgical findings, and postoperative outcomes of the two approaches were compared. Propensity score matching was performed to manage selection bias.

**Results:**

The baseline characteristics, including age, sex, smoking history, presence of myasthenia gravis, pulmonary function, clinical staging, and histological classification were not significantly different. Patients in the robot-assisted thoracic surgery group showed significantly lower fluorodeoxyglucose uptake on positron emission tomography and a smaller preoperative tumor size on computed tomography (*P* < 0.01). Intraoperative blood loss, drainage duration, postoperative hospital stay, and pathological tumor size were significantly lower in the robot-assisted thoracic surgery group (*P* < 0.01). In addition, this group had a significantly higher proportion of cases of early pathological Masaoka stage disease (*P* < 0.01). Overall survival and recurrence-free survival were comparable between the two groups. After propensity score matching, the 56 matched patient pairs demonstrated similar perioperative outcomes, overall survival, and recurrence-free survival.

**Conclusions:**

Robot-assisted thoracoscopic surgery was associated with significantly less intraoperative blood loss, shorter duration of chest drain placement, and shorter postoperative hospital stays than median sternotomy. Robot-assisted thoracoscopic surgery may be considered an effective, minimally invasive approach for early-stage thymoma without impairing survival outcomes.

**Supplementary Information:**

The online version contains supplementary material available at 10.1007/s11748-026-02294-8.

## Introduction

Thymomas are rare neoplasms (0.13 per 100,000 person-years) [1]. Surgical resection is an effective treatment for thymoma, and traditionally, median sternotomy (MS) is utilized. However, it is associated with complications such as intraoperative bleeding, postoperative pain, and surgical site infections [2, 3]. To overcome these limitations, minimally invasive approaches have been explored, including video- and robot-assisted thoracoscopic surgery (RATS). RATS offers several technical advantages, including three-dimensional visualization, enhanced instrument dexterity, and reduced hand tremor [4], and thymectomy performed via RATS has been reported to yield favorable surgical outcomes [5–12]. At our institution, RATS was introduced in 2013 and expanded after insurance coverage in 2018. Nevertheless, because thymoma is a rare disease, robust comparative data between RATS and MS remain limited. We retrospectively analyzed outcomes of early-stage thymoma patients undergoing MS or RATS. To minimize selection bias, analyses were performed after propensity score matching.

## Materials and methods

### Patients

Among the 307 patients who underwent surgery for thymic epithelial tumors between January 2010 and December 2024 at our institution, we excluded 10 patients who underwent video-assisted thoracic surgery (VATS), 60 patients with non-thymoma pathology, and 34 patients with preoperative Masaoka stage III–IV thymoma. A retrospective analysis included 203 patients with Masaoka stage I–II thymoma who underwent surgery (Supplemental Fig. 1).

### Data collection and patient follow-up

The following clinical and pathological variables were collected from our clinical database: age, sex, smoking history, presence or absence of myasthenia gravis (MG), preoperative respiratory function values, maximum standardized uptake value (SUVmax) on positron emission tomography (PET), tumor diameter on computed tomography (CT), clinical Masaoka stage, surgical approach, incision length, operative time, intraoperative blood loss, conversion to open thoracotomy, achievement of complete resection, duration of chest drainage, length of postoperative hospital stay, postoperative complications, pathological tumor diameter, and histological subtype. In addition, the Masaoka staging system [13] was used to evaluate disease stage, and the histological subtype of the tumor was determined according to the World Health Organization classification [14].

Follow-up included blood tests and chest radiography every three months and annual CT examinations during the first two years after surgery, followed by evaluations every six months thereafter. Recurrence was diagnosed based on radiological findings and histopathologically confirmed where feasible. Recurrence and survival data were collected over approximately 10 years.

### Patient grouping

To precisely evaluate the impact of the surgical approach on the clinicopathological factors and prognosis in a cohort of 203 patients, we classified the individuals into either the RATS group (n = 68) or the conventional MS group (n = 135). Additional analyses were conducted in the RATS and MS groups after propensity score matching based on the predefined clinical factors described in the statistical analysis section.

### Surgical approach selection and criteria for the extent of resection

At our institution, MS was primarily indicated for patients with both early and advanced stage thymoma in the past, whereas RATS has been the preferred approach since its introduction. The surgical approach was based on clinical information; following RATS introduction, tumors > 5 cm were more often treated with MS. The extent of resection depends on clinical factors: thymectomy is our standard procedure in patients without MG, extended thymectomy in those with MG or high anti–acetylcholine receptor (AChR) antibody titers, and simple tumorectomy in selected elderly or high-risk patients.

### Details of robot-assisted thoracic surgery (RATS)

Most RATS procedures were performed using a lateral thoracic approach. An 8-mm camera port was placed in the fifth intercostal space, with additional 8-mm robotic ports placed in the third and seventh intercostal spaces. A 12-mm assistant port was positioned dorsally in the seventh or eighth intercostal space. For extended thymectomy, ports were placed symmetrically on both sides, and the procedure was performed bilaterally. When the subxiphoid approach was used, 8-mm ports were placed in the sixth intercostal spaces bilaterally, and an access port integrating both the camera and assistant ports was inserted in the subxiphoid region. For extended thymectomy performed via the subxiphoid approach, additional access ports were placed in the bilateral fourth intercostal spaces (Supplemental Fig. 2).

### Definition of postoperative overall survival (OS) and recurrence-free survival (RFS)

Postoperative OS was defined as the interval between the date of surgery and death from any cause or last follow-up. The duration of postoperative RFS was defined as the time between the date of surgery and the date of recurrence, death from any cause, or last follow-up. Observations were censored at the last follow-up when the patient was alive or lost to follow-up. The data collection cutoff date was October 2025.

### Statistical analysis

Continuous variables were compared using the Student’s t-test, whereas categorical variables were compared using Pearson’s chi-square test or Fisher’s exact test, as appropriate. Survival rates were estimated using the Kaplan–Meier method, and differences in postoperative OS and RFS were assessed using the log-rank test. Although this study primarily aimed to evaluate the precise impact of the RATS approach on clinicopathological factors and prognosis, adjustment for baseline characteristics is necessary for a more detailed analysis. Propensity score matching was applied considering the number of events and explanatory variables. Propensity scores were calculated using a logistic regression model that included the clinical variables of age, sex, smoking history, presence of myasthenia gravis, preoperative pulmonary function, tumor size on CT, and clinical Masaoka stage to adjust for confounding factors between the MS and RATS groups. Matching was performed at a 1:1 ratio using nearest-neighbor matching with a caliper width of 0.2. After matching, balance was evaluated using standardized mean differences. All statistical analyses were conducted using EZR version 1.68 and R version 2.9–1 (www.r-project.org) [15].

## Results

### Follow-up duration

Median follow-up (IQR) was 67.0 (37.25–109.75) months for the overall cohort, 37.0 (11.75–54.0) months for the RATS group, and 92.0 (61.25–120.25) months for the MS group. After matching, the median follow-up duration was 66.0 (36.25–120.0) months for the overall cohort, 37.5 (13.0–54.0) months for the RATS group, and 115.5 (88.5–121.25) months for the MS group.

### Patient characteristics

Of the 203 patients, 68 (33.5%) and 135 (66.5%) were assigned to the RATS and MS groups, respectively. The baseline patient characteristics and tumor-related features of the two groups are summarized in Table 1. In the total cohort, the RATS group had significantly lower PET (SUVmax) values (3.2 vs. 4.1, *p* < 0.01) and a smaller tumor size on CT (3.8 cm vs. 5.1 cm, *p* < 0.01) compared with the MS group. After matching, no significant differences were observed except for in the PET (SUVmax) values. Additionally, at our institution, since RATS became covered by the national health insurance system in 2018, the number of RATS procedures has increased rapidly; consequently, a year-by-year shift in the surgical approach can be observed (Supplemental Fig. 3).

**Table 1:** Baseline patient features in the 2 groups (unmatched and matched)

	RATS (n = 68)	MS (n = 135)	*p* value	RATS (n = 56)	MS (n = 56)	*p* value
Age (years), median (IQR)	65 (51.8–73.3)	61 (52.0–69.0)	0.23	65 (51.0–73.3)	62.5 (51.8–68.8)	0.6
Male sex, n	33 (48.5%)	64 (47.4%)	0.88	25 (44.6%)	26 (46.4%)	1
Current or ex-smoker, n	27 (39.7%)	66 (48.9%)	0.41	25 (44.6%)	26 (46.4%)	0.85
Myasthenia gravis, n	18 (26.5%)	24 (17.8%)	0.19	13 (23.2%)	13 (23.2%)	1
Respiratory function normal, n	54 (79.4%)	96 (71.1%)	0.46	43 (76.8%)	43 (76.8%)	1
PET (SUV max), median (IQR)	3.2 (2.6–3.8)	4.1 (3.2–4.9)	< 0.01	3.2 (2.5–3.8)	3.9 (2.7–4.8)	< 0.01
Tumor size in CT scan (cm), median (IQR)	3.8 (2.6–4.5)	5.1 (3.6–7.1)	< 0.01	3.8 (3.0–4.3)	3.7 (3.0–4.6)	0.67
Clinical Masaoka stage, n			0.34			0.76
I	63 (92.6%)	118 (87.4%)		51 (91.1%)	49 (87.5%)	
II	5 (7.4%)	17 (12.6%)		5 (8.9%)	7 (12.5%)	

Values are presented as number (%) or median (interquartile range).

RATS, robot-assisted thoracic surgery; MS, median sternotomy; PET, positron emission tomography; SUV max, maximum standardized uptake value; CT, computed tomography

*P* values were calculated using the Pearson’s chi-square test or Fisher’s exact test for categorical variables, and he Student’s t-test for continuous variables.

### Operative and postoperative features

The surgical and postoperative outcomes of the two groups are summarized in Table 2. In the overall cohort, the RATS patients had significantly shorter incision lengths than the MS patients (3 cm vs. 20 cm, *p* < 0.01) as well as less intraoperative blood loss (5 mL vs. 114 mL, *p* < 0.01), a shorter duration of chest drain placement (1 day vs. 2 days, *p* < 0.01), and a shorter postoperative hospital stay (4 days vs. 6 days, *p* < 0.01). There were no significant differences in resection type (RATS: simple tumorectomy, thymectomy, and extended thymectomy, 1.5%, 72.0%, and 26.5% vs. MS: 3.0%, 54.8%, and 42.2%; *p* = 0.06), operative time (130.5 min vs. 146 min, *p* = 0.45), postoperative complications of Clavien–Dindo grade ≥ 3 (2.9% vs. 1.5%, *p* = 0.60), postoperative adjuvant treatment (1.5% vs. 1.5%, *p* = 0.41), or recurrence (2.9% vs. 5.2%, *p* = 0.81) between the two groups. These findings remained consistent after matching. In addition, a temporal trend in operative time was observed in the RATS group, suggesting a learning curve effect, with a gradual reduction in operative time as surgical experience increased (Supplemental Fig. 4).

**Table 2:** Operative and postoperative features in the 2 groups (unmatched and matched)

	RATS (n = 68)	MS (n = 135)	*p* value	RATS (n = 56)	MS (n = 56)	*p* value
*Resection type, n*			*0.06*			*0.09*
*Simple* tumorectomy	*1* *(1.5%)*	*4* *(3.0%)*		*1* *(1.8%)*	*3* *(5.4%)*	
* Thymectomy*	*49* *(72.0%)*	*74* *(54.8%)*		*41* *(73.2%)*	*30* *(53.6%)*	
*Extended* *thymectomy*	*18* *(26.5%)*	*57* *(42.2%)*		*14* *(25.0%)*	*23* *(41.1%)*	
Incision length(cm), median (IQR)	3 (3–4)	20 (16–23)	< 0.01	3 (3–4)	20 (17–22)	< 0.01
Operative time(min), median (IQR)	130.5 (97.5–168)	146 (120.5–180)	0.45	129.0 (95.8–166.5)	151.5 (124.8–186.5)	0.51
Intraoperative blood loss(ml), median (IQR)	5 (1–10)	114 (63–188)	< 0.01	5 (1–10)	97.5 (61.3–156.8)	< 0.01
Open conversion, n	1 (1.5%)	–		1 (1.8%)	–	
Drain removal(days), median (IQR)	1 (1–2)	2 (2–2)	< 0.01	1 (1–2)	2 (2–2)	< 0.01
Length of hospital stay (days), median (IQR)	4 (4–5)	6 (5–8)	< 0.01	4 (4–5)	6.5 (6–8)	< 0.01
Postoperative complications (≧Grade3), n	2 (2.9%)	2 (1.5%)	0.6	2 (3.6%)	2 (3.6%)	1
Adjuvant therapy, n	1 (1.5%)	2 (1.5%)	0.41	1 (1.8%)	2 (3.6%)	1
Recurrence, n (%)	2 (2.9%)	7 (5.2%)	0.81	2 (3.6%)	3 (5.4%)	1

Values are presented as number (%) or median (interquartile range).

RATS, robot-assisted thoracic surgery; MS, median sternotomy

*P* values were calculated using the Pearson’s chi-square test or Fisher’s exact test for categorical variables, and he Student’s t-test for continuous variables.

### Pathologic features

Pathological characteristics are summarized in Table 3. The pathological tumor size in the RATS group was smaller than in the MS group (3.4 cm vs. 5.8 cm, *p* < 0.01), and the proportion of pathological Masaoka stage II cases was lower in the RATS group (38.2% vs. 63.7%, *p* < 0.01). After matching preoperative variables, the RATS group continued to show a significantly smaller pathological tumor diameter (3.8 cm vs. 4.2 cm, *p* < 0.01) and a lower proportion of pathological Masaoka stage II disease (39.3% vs. 71.4%, *p* < 0.01).

**Table 3:** Pathologic features in the 2 groups (unmatched and matched)

	RATS (n = 68)	MS (n = 135)	*p* Value	RATS (n = 56)	MS (n = 56)	*p* value
WHO type, n			0.19			0.51
A	5 (7.4%)	11 (8.1%)		4 (7.1%)	7 (12.5%)	
AB	22 (32.3%)	53 (39.3%)		22 (39.3%)	16 (28.6%)	
B1	14 (20.6%)	32 (23.7%)		10 (17.9%)	15 (26.8%)	
B2	22 (32.3%)	28 (20.7%)		15 (26.8%)	13 (23.2%)	
B3	4 (5.9%)	11 (8.1%)		4 (7.1%)	5 (8.9%)	
Unknown	1 (1.5%)	0		1 (1.8%)	0	
Tumor size in pathology(cm), median (IQR)	3.4 (2.2–4.5)	5.8 (4.2–7.2)	< 0.01	3.8 (2.5–4.6)	4.2 (3.4–5.0)	< 0.01
Pathological Masaoka stage, n			< 0.01			< 0.01
I	35 (51.5%)	35 (25.9%)		28 (50.0%)	12 (21.4%)	
II	26 (38.2%)	86 (63.7%)		22 (39.3%)	40 (71.4%)	
III	4 (5.9%)	12 (8.9%)		3 (5.4%)	2 (3.6%)	
IVA	2 (2.9%)	0		2 (3.6%)	0	
IVB	1 (1.5%)	2 (1.5%)		1 (1.8%)	2 (3.6%)	
R, n			1			1
0	67 (98.5%)	134 (99.3%)		55 (98.2%)	55 (98.2%)	
1	1 (1.5%)	1 (0.7%)		1 (1.8%)	1 (1.8%)	
2	0	0		0	0	

Values are presented as number (%) or median (interquartile range).

RATS, robot-assisted thoracic surgery; MS, median sternotomy; WHO, World Health Organization

*P* values were calculated using the Pearson’s chi-square test or Fisher’s exact test for categorical variables, and he Student’s t-test for continuous variables.

### Overall survival and recurrence-free survival

**Fig. 1:**
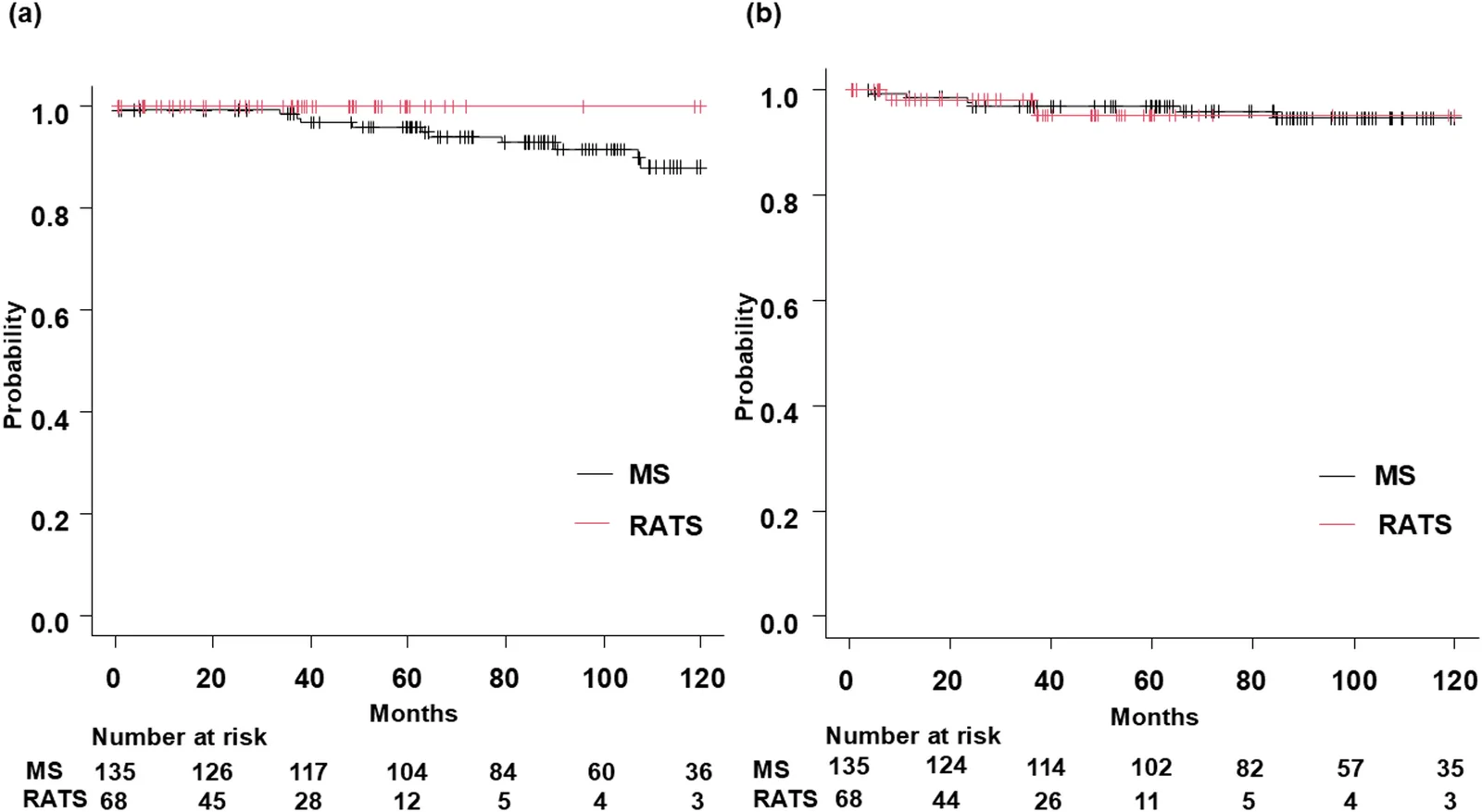
Kaplan–Meier curves for the MS and RATS groups **a** Overall survival rates in the two groups; the 5-year overall survival rate was 95.9% in the MS group and 100% in the RATS group. **b** Recurrence-free survival in the two groups; the 5-year recurrence-free survival rate was 96.9% in the MS group and 95.2% in the RATS group. MS, median sternotomy; RATS, robot-assisted thoracic surgery

**Fig. 2:**
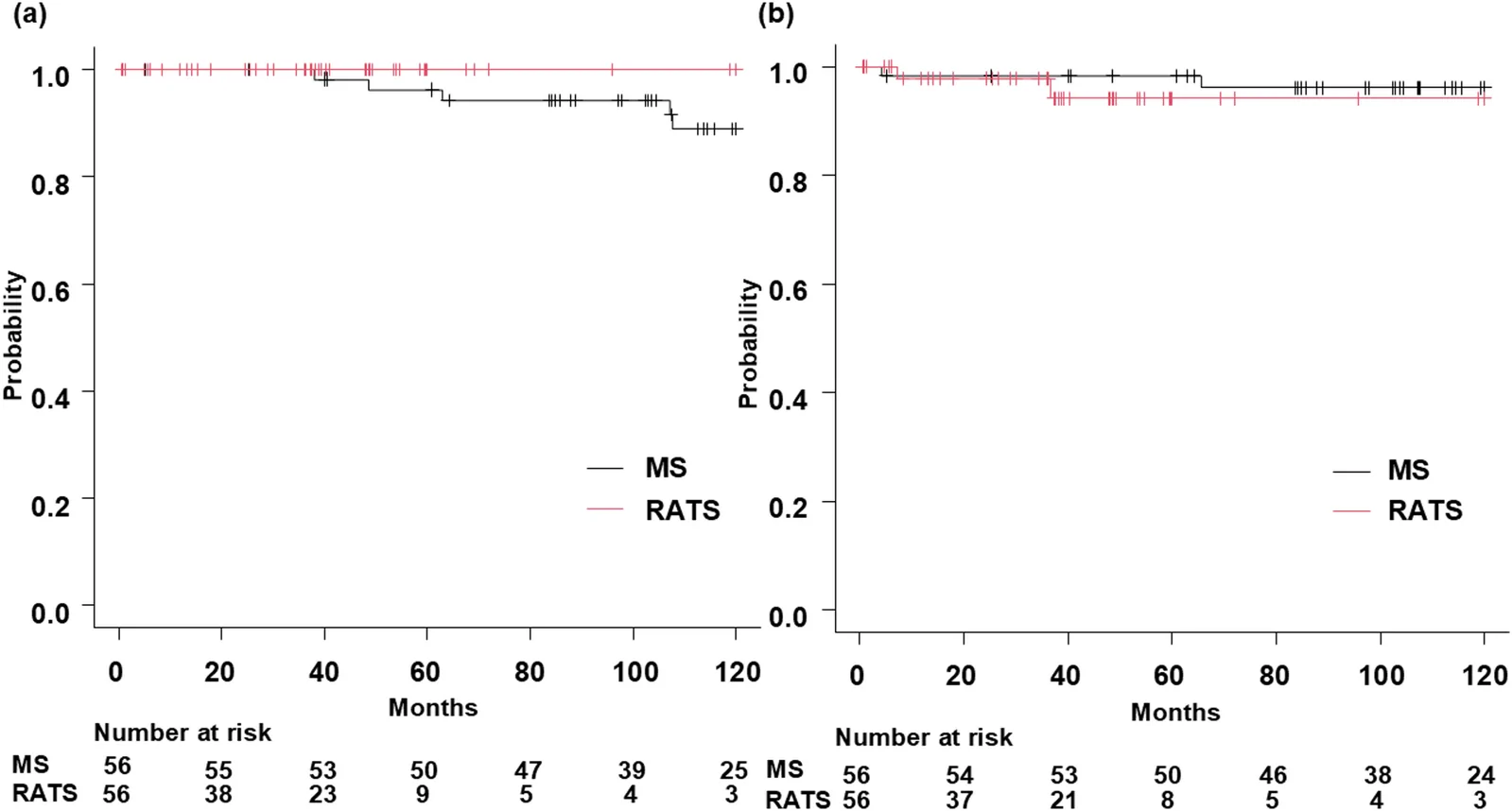
Kaplan–Meier curve for the MS and RATS groups after propensity score matching. **a** Overall survival rates in the two groups; the 5-year overall survival rate was 96.2% in the MS group and 100% in the RATS group. **b** Recurrence-free survival rates in the two groups; the 5-year recurrence-free survival rate was 98.2% in the MS group and 94.2% in the RATS group. MS, median sternotomy; RATS, robot-assisted thoracic surgery

The OS and RFS of the two groups in the overall cohort and after matching are shown in the Kaplan–Meier curves in Figs. 1 and 2, respectively. In the overall cohort, the 5-year OS rates were 100% in the RATS group and 95.9% in the MS group, and no significant difference in OS was observed between the two groups according to the log-rank test (*p* = 0.15). The 5-year RFS rates were 95.2% in the RATS group and 96.9% in the MS group, respectively, with no significant differences in RFS between the groups based on the log-rank test (*p* = 0.84). After propensity score matching, the 5-year OS rates were 100% in the RATS group and 96.2% in the MS group, respectively, with no significant difference in the OS between the two groups on the log-rank test (*p* = 0.26). Similarly, the 5-year RFS rates were 94.2% in the RATS group and 98.2% in the MS group, respectively, and the log-rank test showed that there was no significant difference in RFS between the two groups (*p* = 0.52).

## Discussion

Surgical approaches for treating patients with thymic epithelial tumors have evolved over time. Compared to MS, which has long been considered the standard approach, VATS has been reported to be less invasive and associated with faster postoperative recovery [16, 17]. More recently, RATS, which is less invasive and results in quicker postoperative recovery compared to MS, has been increasingly adopted because it allows magnified visualization and precise surgical manipulation [7, 10, 11]. As mentioned above, previous studies have predominantly compared MS with VATS or RATS in thymic epithelial tumors. Although previous studies have compared MS with VATS or RATS, and some have included all three approaches or meta-analyses [4, 18], many analyses incorporated benign thymic disease or thymic carcinoma. RATS for advanced-stage thymoma remains controversial; therefore, at present, its application is restricted to advanced cases of stage III and IV and still consider MS the standard surgical approach for these patients. This study evaluated perioperative outcomes and long-term prognosis in what is, to our knowledge, the largest cohort comparing RATS and MS for early-stage thymoma (Masaoka stage I–II).

Regarding patient characteristics, the RATS group had significantly smaller tumor diameters than the MS group, as reported in previous studies [3, 6, 10, 12, 19]. Although some reports have shown that differences in tumor size remain significant even after propensity score matching [19], in the present study, no significant difference in tumor diameter, as determined by CT, was observed between the two groups after propensity score matching (3.8 cm vs. 3.7 cm, *p* = 0.67). However, even after matching, the pathological tumor diameter was significantly smaller in the RATS group compared with that in the MS group (3.8 cm vs. 4.2 cm, *p* < 0.01). The MS group had a higher proportion of tumors with more rapid growth than the RATS group. Both before and after propensity score matching, the RATS group included a higher proportion of patients with earlier pathological Masaoka stages and exhibited lower PET SUVmax than the MS group. These findings suggest selection bias, which may be due to more advanced cases being more likely to be treated using the MS approach. With respect to perioperative and postoperative outcomes, RATS was associated with significantly less intraoperative blood loss and a shorter time to chest drain removal than MS, which is consistent with the findings of previous studies [3, 10–12, 18, 19]. These advantages translated into shorter postoperative hospital stay in the RATS group, likely reflecting the minimally invasive nature of the robotic approach and its contribution to early postoperative recovery. Regarding operative time, some previous studies have reported longer operative times for RATS compared with MS [7, 10, 18, 19]. However, in the present study, no significant difference was observed between the two groups, and the median operative time was shorter in the RATS group than in the MS group (130.5 min vs. 146 min, *p* = 0.45). This finding is consistent with prior meta-analysis [4]. A learning curve likely contributed to the reduction in operative time [10, 20]. The RFS was comparable between the two groups, but a trend toward higher OS was observed in the RATS group compared with that in the MS group, although the difference was not statistically significant. A similar trend was reported by Marulli et al. [19]. However, this is most likely attributable to differences in follow-up duration between the two groups, and accurate assessment of long-term outcomes in the RATS group remains difficult.

The strengths of the present study include that, to the best of our knowledge, the patient cohort is the largest to date among other studies comparing RATS and MS for early-stage thymoma, as well as the use of propensity score matching, which enabled a comparison between the two groups without a significant difference in tumor diameter on CT imaging.

This study had several limitations. First, this was a single-center retrospective study. However, in multicenter studies, procedural variations may exist even within the same surgical approach, and long-term prospective follow-up is not realistic in the current clinical environment where treatment techniques evolve rapidly, resulting in a limited number of cases. Second, as RATS was introduced more recently than MS, the median follow-up period was longer in the MS group than in the RATS group. These differences in follow-up duration may have influenced the OS and RFS outcomes. Third, although this study analyzed patients with early-stage thymomas undergoing different surgical approaches, the potential for selection bias cannot be completely ignored. In the overall cohort, patients with larger tumors tended to undergo MS. To minimize this limitation, propensity score matching was performed to create two comparable groups. However, the possibility of a residual, unevaluated selection bias remains.

## Conclusion

RATS was associated with less intraoperative blood loss, shorter drainage duration, and shorter postoperative hospital stays than MS. No significant differences in survival outcomes were observed; therefore, RATS may be considered an effective minimally invasive approach for treatment of early-stage thymoma. Further studies are warranted to validate these findings and long-term survival outcomes.

## Supplementary Information

Below is the link to the electronic supplementary material.

Supplementary Material 1

Supplementary Material 2

Supplementary Material 3

Supplementary Material 4

## Data Availability

The data will be shared on reasonable request to the corresponding author.

## Abbreviations

RATS: Robot-assisted thoracic surgery
MS: Median sternotomy
FDG: Fluorodeoxyglucose
PET: Positron emission tomography
CT: Computed tomography
OS: Overall survival
RFS: Recurrence-free survival
VATS: Video assisted thoracic surgery
CI: Confidence interval
